# Use of Creative Writing to Develop a Semantic Differential Tool for Assessing Soundscapes

**DOI:** 10.3389/fpsyg.2018.02698

**Published:** 2019-02-05

**Authors:** David Welch, Daniel Shepherd, Kim Dirks, Mei Yen Tan, Gavin Coad

**Affiliations:** ^1^School of Population Health, The University of Auckland, Auckland, New Zealand; ^2^Department of Psychology, Auckland University of Technology, Auckland, New Zealand; ^3^Bay Audiology Ltd., Auckland, New Zealand

**Keywords:** soundscape, questionnaire, qualitative methodology, quantitative methodology, psychometric

## Abstract

Exploring our understanding of soundscapes to understand why and how sound impacts people is important. The aim of this study was to develop a short quantitative questionnaire that would use terms generated by creative writers to assess people’s experiences of a soundscape. This process may provide different items for the questionnaire and thus, potentially, different dimensions or fuller definitions of dimensions that have already been identified. In the preliminary phase, a group of people identifying themselves as good writers listened to recordings of natural, traffic, and human sound environments and wrote about their impressions and responses to each. Qualitative analysis was used to extract themes from the writing. These themes were identified by key words, and scalar items were developed to form a short 17-item questionnaire. The questionnaire was administered to 228 people in Auckland City, New Zealand, with participants recruited from city streets and in a central-city park. Respondents were comfortable to use the questionnaire. Factor analysis revealed patterns of responding with five dimensions: Calming, Protecting, Hectic, Belonging, and Stability. There were correspondences between these and others previously reported in the literature, as well as differences. The use of items derived from creative writing provided interesting insights into the soundscape, including spirituality, the sense of time passing, and physical wellbeing. The park soundscape was measurably better than the street soundscapes on all dimensions, and streets with less vehicular traffic tended to be experienced as more Calming and Protecting, and less Hectic. This implies that there is validity in the scales generated. In future, it would be valuable to test the questionnaire in more varied environments, to add greater variability to the soundscapes.

## Introduction

Sound has been shown to impact on people’s physical and mental health (Basner et al., 2014), as has the loss of the access to sound in severe or profound hearing loss (Guitar et al., 2013). Nonetheless, many people appear to lack awareness about the importance of sound and hearing so that troubling noise is widespread in our society (Welch et al., 2013), and noise-limiting or hearing-health programs are fraught with difficulty (e.g., Reddy et al., 2012). The concept of the soundscape may be a useful way to understand and thus communicate with other people about the effects of perceived sound in order to improve our societies’ sound environments (Schafer, 1977; Andringa et al., 2013; Steele et al., 2015). The aim of this study was to improve our understanding of it and to add to the development of an instrument to measure it quickly and effectively.

One way of accessing a person’s representation of the world based on their sensory experience is through language (Raimbault, 2006), though other approaches [e.g., comparison to music (Botteldooren et al., 2006)] have been considered. Qualitative approaches have the capacity to delve deeply into people’s narratives for meaningful descriptions of what they perceive. Compared to quantitative data, qualitative data are rich. However, it can be more difficult to make comparisons between qualitative measures and can be harder to obtain quick and accurate responses at a population level, especially when seeking responses from less educated or literate people. Another issue with qualitative descriptors of soundscapes is that they may be limited by a person’s vocabulary and ability to express themselves using language. Given that most people understand more words than they will actually use (Laufer, 1998), providing people with a set of descriptors which can be rated may allow them to report on experiences for which their active vocabulary would be insufficient but for which their passive vocabulary compensates.

A tool that has been used in the context of a soundscape is the semantic differential scale (Osgood et al., 1957; Kang and Zhang, 2010; Cain et al., 2013). The approach takes the form of a set of adjectives, and requires the respondent to select a number between two poles of a continuum (e.g., pleasant vs. unpleasant). An advantage of this method is that the same subjective attributes may be compared between different locations quantitatively. Some terms are described as ‘denotative,’ or referring to aspects of the sounds being experienced (e.g., fast/slow); and others are described as ‘connotative,’ or referring to a person’s response to the sounds (e.g., calming/agitating). Parallel terms, more suited to soundscape research, have been used, with ‘descriptive’ for denotative and ‘affective’ for connotative. The challenge is to find terms that are easy to understand but which also allow a respondent to express the subtleties of their experience of a soundscape (Raimbault, 2006).

The semantic differential method presents opposing soundscape descriptors on a scale which is considered to be unidimensional (Osgood et al., 1957). On the other hand, previous research into the cognitive representations of soundscapes and their descriptors suggests that there may be heterogeneity in the interpretation of the lexical items used and thus of the determinants of respondents’ choices (Dubois et al., 2006). In other words, while the semantic differential may be a useful quantitative method for the analysis of experiential factors, it must be borne in mind that it cannot represent an objective or absolute measurement of a soundscape attribute. A superficial appearance of consensus which may occur is that respondents will use a set of terms presented to them, but there may be variation in the meaning of those terms for each person. The process of developing the semantic markers is thus crucial in providing respondents with acceptable and clear responses, and the introduction of different markers may potentially provide the opportunity to present new ways of perceiving the world.

Factor analysis, and the related principle components analysis, has been used extensively with semantic differential scales in the soundscape literature (e.g., Kang and Zhang, 2010). Factor Analysis combines a statistical approach with subjective judgment (Tabachnick and Fidell, 1996). It aims to simplify people’s responses to many semantic differential scales by identifying the underlying perceptual/emotional dimensions (factors) that influence the original responses. To do this, it measures correlations between responses to different items, and where several items correlate to a reasonably high degree, a factor is generated. The subjective exercise is the ‘naming’ of factors based on aspects of the items contributing most to each. The naming exercise relies heavily on the choice of word made in forming the semantic differential scales. Furthermore, in reducing several items to one name that encompasses all of their meanings, it relies on the minds and vocabularies of the researchers to capture the commonality appropriately. Factor analysis cannot, of course, look outside the set of original items and the responses to them so it searches for correspondences within a closed set and cannot be used to comment on the extent to which a particular approach has captured the true variance in people’s thoughts.

As such, the process of deciding upon the original set of items is crucial and a range of approaches has been used. For example, one approach has been review of the literature on sound descriptors and rendering down of a larger list into twelve items researchers perceived to be most appropriate for the task along with pilot testing (Berglund and Nilsson, 2006). Another approach used a list of 116 items that were based originally on terms extracted from interviews about photographs, with reference to sound-relevant terms and consensus from a group of experienced listeners (Axelsson et al., 2010). Others have used a combination of terms derived from literature and items decided upon by the researchers as relevant to the environment being studied (Kang and Zhang, 2010). These approaches are well-considered and have generated quite similar sets of items, each of which has face-validity as a potential descriptor of a soundscape. A class of approach that has been applied to gather data relevant to the soundscape is interviewing with qualitative analyses (Liu and Kang, 2016), but it has not been reported as a preliminary stage in the consideration of items for semantic differential scales. An advantage of using such an approach would be that a reduced battery of questionnaire items could be used. These items would be based on the themes identified in the qualitative research, and would therefore provide a good structure for the soundscape while also reducing the length of the questionnaire.

On the basis of this, it is desirable to establish a set of dimensions that people tend to use generally when making judgments about a soundscape. Some progress has been made in this direction (Davies and Murphy, 2012). Furthermore, a theoretical basis, rooted in evolutionary psychology, has been proposed to explain why these dimensions might be common for people across cultures (Andringa and Lanser, 2013; van den Bosch et al., 2018). Essentially, this theory suggests that the environment might be perceived in terms of two factors: whether it is pleasant for the organism, and whether much is happening. The two concepts may be regarded as orthogonal in that an environment can be rich (pleasant and eventful), dangerous (unpleasant and eventful), calming (pleasant and uneventful), or boring (unpleasant and uneventful). Two dimensions, Pleasantness (emotional valence) and Eventfulness (vibrancy), have been identified in several soundscape studies (De Coensel and Botteldooren, 2006), and it has been suggested that these might be seen as two basic dimensions of soundscapes (Davies et al., 2013; Aletta et al., 2016). These dimensions may be seen to reflect the basic dimensions of human mood, as expressed in earlier research (Russell, 1980).

The dimensionality of the soundscape may be more complex, and has varied across studies. There are many possible reasons for this, including differences in the sound environments and the methods used to collect responses. A four-dimensional model derived from factor analysis: “Relaxation,” “Communication,” “Spatiality,” and “Dynamics” has been developed to account for urban soundscapes (Kang and Zhang, 2010). Research using only affective (i.e., connotative) semantic differential attributes (e.g., “pleasant” and “calm”) and not descriptive (e.g., “loud” and “sharp”) found three components: “Pleasantness,” “Eventfulness,” and “Familiarity” (Axelsson et al., 2010). In other research, two principal components: “Calmness” and “Vibrancy,” which may be seen to parallel Pleasantness and Eventfulness, were identified (Cain et al., 2013). Other work has identified the concept of “Restorativeness,” the sense that a soundscape helps people to recover from tiredness or malaise (Payne, 2013). Furthermore, the concept of “Appropriateness” (a sense that the soundscape is right for the place in which it is experienced) has been considered as an aspect of sound environments which should be considered in terms of soundscapes (Axelsson, 2015). Each of these dimensions has been shown to have some reliability, and yet they vary and differ between studies. The differences may arise partly due to variations in the sound environments or stimuli used in different studies, but they may also depend upon observers’ ability to express their perceptual experiences. The more varied the response options that can be provided, the more detail may be understood about human soundscapes.

It is likely that there is commonality in the human experience of soundscapes (Brown et al., 2011), so it may be possible to generate a short and quantitative measure to capture this. A key issue is the need for a good set of terms to allow people’s responses to the soundscape to be captured, since if a concept is missing, there will be no way to detect its absence. Our approach had three main stages:

(1) We asked people with high active vocabularies and an interest in written expression to write about their responses to three different sound environments.
(2) We analyzed these writings for themes that were present in the responses.
(3) We used these themes in a short questionnaire that we administered to a small sample of people in Auckland City.

The rationale for choosing literate people was based on the principle described above that people with limited active vocabularies will typically have larger passive vocabularies. Since people may be induced to draw upon their passive vocabulary when prompted, and a semantic differential questionnaire is essentially a set of prompts, the approach seemed reasonable. We ran exploratory analyses on the results to see whether the approach had produced potentially useful data. In particular, we were interested to see whether members of the general public could use the questionnaire to describe their perception of the sound environment and their responses to it quickly and easily.

## Phase 1: Qualitative Study

There were two phases to the research. Phase 1 involved recruiting literate people with an interest in descriptive writing and/or sounds. These participants wrote about their perceptions and responses to three different sound environments, and their writing was analyzed using thematic analysis (Braun and Clarke, 2006). A questionnaire was developed based on the themes identified. Phase 2 was a piloting of the questionnaire in a sample of people in real sound environments in Auckland City, New Zealand. The research was approved by the University of Auckland Human Participants Ethics Committee: Approval number 8150.

### Materials and Methods

#### Participants

Twenty-five adult participants aged 20–38 years (Mean = 25.04, *SD* = 4.71) participated; 52% were male (*n* = 13). Recruitment was through advertisement in the form of posters, electronic flyers, and social media. It was desirable to attract participants who would be willing and able to provide rich written descriptions of their responses to different sound environments, so advertising was targeted to students in creative-writing courses at the university. All participants had hearing thresholds of better than 20 dBHL in their better ear for all tested frequencies.

#### Procedure

Three recordings were selected on the basis that they represented sound environments dominated by sounds of nature, humanity, and technology. These classes of environment have previously been shown to produce differences in the types of descriptor used for the soundscapes arising from them (e.g., Axelsson et al., 2010). They were purchased from a database of environmental sound recordings at www.shockwavesound.com. The three soundscape recordings were in 5.1 surround sound AC3 (Dolby digital) file format, and brief descriptions of each are as follows:

(1) Traffic: Road traffic noise recorded at a town junction. Cars, mopeds, motorcycles, and occasional buses accelerating past in all four directions with some distant voices.
(2) Human: Crowded pedestrian street in town. People walking by in all directions, distant sound of children playing.
(3) Nature: Light surf with small birds chirping and tweeting to the front and rear.

The original recordings were looped to extend the presentation duration using Audacity^®^ 2.0.0. After this processing, the participants were presented with recordings of sound environments that lasted between 19 min 27 s and 26 min 41 s. The recordings were crossfaded over 3 s to avoid sudden changes. The presentation order was randomized across participants, and soundscape assessment conducted in a sound attenuating chamber 2.21 m wide and 2.48 m long.

A Sony 6.1 surround speaker system consisting of left (L), centre (C), right (R), left surround (Ls), right surround (Rs), centre back (Cb) speakers and a subwoofer (Sub) was used. The speaker system was treated as a 5.1 surround system, and no input was received at the Cb speaker for the 5.1 soundscape recordings file format. All speakers were facing the listener and mounted on adjustable stands, with the exception of the subwoofer. The speakers were amplified with a Sony digital audio/video (Model STR-DG500 6.1 Channel) amplifier.

The 6.1 surround system was set up as follows:

(1) The C speaker was positioned straight ahead of the listener at 0° azimuth.
(2) The L and R speakers were positioned at each corner of the front of the booth, approximately 45° left and right, respectively, to the horizontal. The speakers were raised slightly above ear level.
(3) The Ls and Rs speakers were positioned at each corner of the back of the booth, approximately 45° left and right, respectively, to the midline. The speakers were aligned at ear level.
(4) The non-functioning Cb speaker was positioned directly behind the listener.
(5) The Subwoofer was positioned at the front between speakers C and L.

A calibration spot approximately 150 cm from each of the L, R, Ls, and Rs speakers to the middle of the room was marked with masking tape. A comfortable chair on which participants were seated was positioned over the calibrated spot, and a large table was situated in front of the chair where the amplifier and a laptop were placed.

Sound recordings were delivered through the surround sound speakers using VLC media player on a Macbook. The coupling of the laptop with the amplifier was carried out with a Creative Labs Sound Blaster THX^®^ TruStudio Pro external USB soundcard and an optical audio cable.

Output levels of the three sound environment recordings were calibrated using a Brüel and Kjær Hand-Held Analyzer Sound Level Meter (Type 2250) with a ½ inch microphone. The sound level meter was mounted on a Manfrotto 804RC2 tripod at participants’ ear level when seated over the calibrated spot.

The average sound pressure level (SPL) of traffic sounds was set to 75 dBA (LAeq, 4 min). We based this on an estimate which indicated that the average SPL of traffic noise taken from major Australian cities ranges between 55 and 75 dB (Austroad Facts, 2000). The upper limit of this range was taken because the intersection was very busy and this level sounded appropriate to the researchers. On the same basis of the researchers’ subjective experience of the sound (what “sounded right”), the average SPL of human sounds was set to 65 dBA (LAeq, 4 min), and the average SPL of nature sounds was set to 55 dBA (LAeq, 4 min).

Each participant was seated and briefed about the context of the sound environments before commencement of each recording. While listening to each recording, participants were instructed to write about their soundscape experience. Participants were given the option of manually writing their responses with pen and paper or typing on a laptop, but all preferred the latter. A blank Microsoft Word document was created headed with an open-ended question:

“Please describe the soundscape you have just heard, and the feelings, emotions, and impressions it may have evoked in you (for example, positive or negative reactions you may have)”

Participants were instructed to write as freely as possible in response to the question. For each of the soundscape recordings, participants were informed that the minimum writing time was 8 min. However, they were encouraged to write as much as they could, and allowed as long as they required. A count-up timer was set up in the top right-hand corner of the laptop screen to notify participants when 8 min had passed.

During the experiment, the researcher waited outside the booth in order not to interfere with the soundscape experience and to preserve the anonymity of participants’ writings. Participants were asked to leave mobile phones outside the booth. The lights of the sound-proofed booth were dimmed during the experiment.

#### Qualitative Analyses

Each participant wrote in response to each of the three sound recordings. Participants’ subjective writings in response to the open-ended question were analyzed using NVivo Software. A thematic analysis of the writings was conducted, and a set of themes and concepts within the data was identified. These categories were organized in a hierarchical manner, illustrating the emergence of more specific themes from general concepts. Coding was conducted by authors MT and DW, who worked both independently and together in order to propose and clarify themes, and achieve consensus.

A thematic analysis approach was used (Braun and Clarke, 2006). The coders read the writing and described themes that they felt underlay each passage. A ‘passage’ is not clearly defined, but is described by the coder in the process of analysis according to their understanding of meaning, and quoted as appropriate (see below). Furthermore, a given passage may potentially be coded as expressing multiple themes, and a theme can be expressed many times or just once: the frequency is not relevant since no sampling frame or specific, *a priori* definitions are used. The analysis seeks to discover a hierarchical structure whereby the themes expressed can be described. The hierarchy is a system of general themes and subthemes that allows the coders to perceive a pattern and to extract elements of meaning. It is thus a subjective approach, and uses the coder’s mind as the lens for understanding the themes underlying what is written. It is possible to approach the data with a pre-conceived theory, and look for themes that are relevant to the components of that theory. In this case though, themes were allowed to emerge from the data without an explicit theoretical stance. However, both coders were aware of themes/descriptors that had been used in previous research into the soundscape, so this may have influenced our thinking. Themes were labeled based on what the coders believed to be the soundscape feature that underlay the writing.

More specifically, at the highest level, we classified responses into those which were descriptive of qualities of the sound environments, and those which were relevant to the response generated in the person while experiencing the soundscapes. In the qualities of the sound environment were responses that reflected: temporal qualities of the sound, which contained sub-themes related to (1) pace (leisurely versus fast) and (2) patterning (with concepts like rhythm or predictable patterns versus irregular or unpredictable sounds); (3) the overall level of the sounds; (4) the extent to which the sound environment was described as clear versus blurred or disorderly; (5) the complexity of the sound environment; (6) the spatial qualities, including sub-themes relating to vastness as opposed to congestion; (7) the sense of tonality or harmony versus discordancy or harshness; and (8) the stability as opposed to variability of the sound environment. The responses to the soundscape were classified into three general areas: health, physical responses, and responses of the *psyche.* In this latter category, we drew on its usage in reference to cognition, as well as the concept of the spirit or soul. Health responses included themes relating to (9) wellbeing, with ideas like wholesomeness versus a sense of affliction; (10) stimulation or arousal versus hypnosis; and (11) stress, including distress and anxiety versus a sense of relaxation. Physical responses included themes relating to (12) safety versus feeling threatened and fearful; and (13) comfort including ideas like contentedness versus having a desire to escape. The responses of the psyche were divided into those which were either cognitive or soulful. Within the cognitive set of themes were those related to (14) cognitive load or burden as opposed to a feeling of being refreshed; and the sense of (15) familiarity or usualness versus novelty. The soulful themes included feelings of (16) connection to the soundscape, and (17) a spiritual sense of being uplifted versus being oppressed. In these different themes, there were statements that supported positive and negative aspects, and the ideas that were expressed helped to develop anchor points for the scales generated from each theme. The numbering in the foregoing text are to allow the reader to see the eventual themes that emerged and were included as scales in the questionnaire; these are explained more fully with supporting quotes in the “Results” section below.

### Results

Themes fell into two general classes: themes relating to the perceptions of the sounds themselves, and themes about the feelings and impressions that were evoked by the soundscapes. The distinction was not always clear, but we presented the themes according to this. For example, the sound of an internal combustion engine presented at a high level may be perceived as loud, and this may make a person feel disturbed. In our analysis, the component of the report, ‘loud,’ was treated as a report about the qualities of the sound and the component, ‘disturbing,’ was treated as a report about the person’s deeper feelings and emotions. We acknowledge that ‘loudness’ is a perceptual quality, and may contain the sense of being disturbing.

#### Qualities of the Sound

The impression of loudness was identified as a theme, especially in the exposure to traffic sounds:

“The blood that runs in the city’s veins is harsh and loud…”

“Lots of loud noise, motor noises are not the most relaxing sounds- especially motorbikes. Constant noise- there may be quieter moments, but there is always background noise, and the quieter moments do not last long.”

It can be seen from the second quote that the sense of loudness/quietness was, as might be expected, seen as a continuum from loud through quieter stimuli. This quantitative aspect seemed to be present generally and accorded with our use of bipolar scales in the design of the questionnaire.

A sense of pace, particularly speed was perceived in the sounds. Again, this was referred to as if it were a continuum, and manifested as a sense of the temporal combined with the emotional. In other words, it suggested that the soundscape included a sense of the passage of time and that this was intertwined with a need to act in a manner consistent with the temporal imperative. For example, urgency is conveyed in these quotes from the traffic and pedestrian sound environments, respectively:

“The brakes stop abruptly, signifying that time is short, and nobody has time to spare in their busy schedule. Nobody has time to spare, everyone minding their own business.”

“A sense of urgency followed by a wave of panic fills the air… Everything is moving so fast in this town, like someone or something is coming.”

“Hustle. And. Bustle. Not in the good way. Someone get me out of here.”

In the last quote, the implication is that speed, ‘hustle and bustle,’ is sometimes a pleasant thing but that it can also be unpleasant as in the case of the busy traffic. At the other end of this continuum was a sense of leisureliness and the gradual nature of processes in the sound environments associated with a change in the pace of time:

“Time slows down to an almost standstill.”

“The water’s course over the stone will erode it. The stone fades just as we do, just a little slower. One must appreciate the beauty that must all fade away. One feels, too, that the sea is hidden behind a verdant curtain; tall trees at the border of the garden perhaps, or simply thick growth on shorter flora. One catches glimpses of the fast passing of waves and the slow passing of stones through this curtain, just as one does of the world, of life.”

The idea that the sound environment provides cues about the slow erosion of stone due to the action of water provides a compelling sense of how a sound environment may evoke the quality of slowness. And the relationship between the soundscape and time was not necessarily straightforward; variations in the speed of different aspects of it could be part of the reason why a sound environment was pleasant:

“These sounds of nature to me are so peaceful and different. They change but stay the same. The surf is always rushing but it’s the intensity that changes. The birds tweet but the rhythm and speed changes.”

A sense of the clarity of sounds seems to reflect the apparent signal-to-noise ratio for interesting sounds in each environment. In some soundscapes, sounds were distinct with a clear source:

“It is not difficult to separate the sounds of the ocean versus the bird calls, but if I close my eyes the sounds start to merge into an overall panorama of peaceful noise that is just very pleasant to listen to.”

Whereas in others they were not:

“People’s footsteps and voices are drowned out by the constant hum of traffic.”

“I can hear many voices. A hairball of voices. A clogged pipe system of voices. An imperfect spaghetti bowl of vocal chords tied together and spiraling inefficiently. A sound, assassinated.”

Interestingly, in the first quote, the clarity is present but the participant was happy to allow the sounds to merge. In the second quote, the sense is that the human sounds are overwhelmed and lost in the noise from the traffic.

Part of the descriptions of the sound environments seemed to refer to their complexity or lack thereof, and neither was intrinsically good or bad; sometimes the complexity seemed a violent tangle:

*“… there are more people speaking at once and several other background noises competing against each other for attention.”* *“There’s too many things happening (like different people’s conversation) and it gets distracting”*

On the other hand, for some, the lack of complexity in the natural soundscape could be seen negatively:

“I feel as if I would be easily bored as there aren’t many new sounds (just birds and waves crashing around).”

There was an awareness of spaciousness associated with some sound environments:

“There is also a sense of a large expanse of the ocean, the beach (perhaps) and because there are birds there would be places that they can fly off away to.”

While others conveyed a sense of crowding, proximity, and congestion:

“Sounds busy and congested. Felt a bit tight and restricting at first, almost stressful initially.”

“There is a distant clanging of cutlery, babies crying… everything that exists in a densely populated space.”

A tonality was perceived. In some sound environments it was harmonious:

“The chorus the sea sings as the wind encourages its wave to crash. What other melody can compare to that?”

Whereas others were discordant, jangling, or harsh:

“There are a range of voices of different pitches that I can hear. The higher pitched voices – children and women – seem easier to pick out as they move around. But occasionally a man’s voice stands out. Sounds such as babies crying are suddenly quite startling.”

A sound environment could be stable and unchanging or varied and changing. Stability did not seem to refer to the individual acoustic components, but rather that there was a constancy to the various components of the sound environment:

“The surf is always rushing but it’s the intensity that changes. The birds tweet but the rhythm and speed changes. You feel like you could sit for hours and never tire of hearing the same sounds over and over.”

A pattern was observed in some soundscapes which had predictability:

“The ocean waves are rhythmic and predictable and quickly become part of a soothing background.”

“I can hear a low thunderous rumble almost continuously in the background, which seems to stay at about the same volume throughout. At times this rumble seems to almost pulsate and feel sort of rhythmic.”

But others were irregular and unpredictable:

“Sounds such as babies crying are suddenly quite startling and immediately noticeable, as are short claps”

#### Feelings and Emotions

Like the sound qualities above, the internal feelings that people expressed as resulting from the sound environments generally followed a pattern of having two poles with intermediate states.

Stimulation was experienced as a result of perceiving the soundscapes. At one end of the spectrum was the effect of arousing people. This could be pleasant, invigorating and exciting:

“I enjoy myself. It’s not every day I get to go to such a busy and exciting place. The clatter of shoes, the banter of people, the merchants having welcoming and, sometimes sly, smiles, it’s to be an eventful afternoon.”

Or else the level of stimulation could seem too much:

“I can sense urgency in the air. My heart is starting to race. [… ] Why can’t I relax? I need to breathe.”

At the other pole from arousal was a sense of feeling soothed or calmed by the sound:

“I like the sound of the ocean waves. The repetitive white noise has a kind of calming, hypnotic effect that could put me to sleep at night.”

There was a theme reflecting a perception of connection to the environment and the things in it:

“But this is no kind of loneliness, for there is the connection with the greener beings.”

“There is synchrony between the birds and waves. They sing to me with love. Each wave, though far, seems to lap playfully at my feet like a playing child, wanting me to come and join. Welcoming. Appreciating. I have nothing more to think. My body unwinds and settles into this natural rhythm.”

   *“I also feel almost a sense of belonging – I am most familiar with the sounds of a busy inner city and foot traffic. I feel like I’m in my comfort zone and I know where I’m going.”*

And on the other hand, the soundscapes could produce a sense of alienation:

“I feel isolated from them. This is their everyday [… ] I feel invisible, lost, lonely even. It’s as though they are alien to me.”

“I feel squashed and I don’t feel quashed. Just… removed. I am observing, remember, and I am watching people at the game of going.”

“There is also a sense of isolation even though it sounds like there are people around me and I hear voices and people slamming car doors. I know that I am not alone because there are people driving the vehicles and people walking on the street – but it seems everyone in the scene is busy focusing on their own lives and their own actions and almost ignoring me… This makes me feel rather alone and isolated”

The sound environments caused people to feel stressed:

“It’s only when I’ve stopped and listened to it that I realize how harsh and stress-inducing it is. Seems to be a tiring environment to be in – I’m really craving for some quiet time in a park or at home from all the noise.”

Or to feel relaxed

“I feel at rest, worrisome thoughts I may once have had are long forgotten, and I pause to enjoy the sound of nature.”

“It feels great to be listening to this. I can literally feel my body relax… my muscles being less tensed, my mind slowing down in thoughts. I feel like I just find somewhere to lie down and rest, maybe read a book, enjoy the breeze, hear the birds sing. Ahh… it’ll be such a wonderful experience.”

A sense of familiarity is evoked by some soundscapes:

“I feel that this is a very normal, everyday environment to be in.”

“The whole thing is busy, bristling with noise and bustling with the familiar sounds of modern life.”

And this may either put people at ease:

“I feel like I’m in my comfort zone and I know where I’m going.”

Or it may seem unpleasant, dull and boring:

“The racket is almost unbearable. Although all too familiar.”

“We are all following a pattern designed by something larger than ourselves, all moving, busy ants picking up a little lump of the bigger sugar pile, picking it up, carrying it and dropping it somewhere, only to be picked up and moved again by a fellow ant. I want out. This is not me.”

The sense of a Cognitive Load or burden placed on or removed from the mind by experiencing the soundscapes was suggesting. Sometimes this load was heavy and crushing:

“Too many things, too many noises surround me it’s hard to hear your own thoughts.”

“I feel smothered by the constant hustle and bustle. Mind feels saturated with thought trying to take in everything but unable to hear my own thoughts.”

Whereas the removal of the load could be refreshing:

“I can feel my mind coming alive, as if a blanket of responsibility that has been smothering me has been removed.”

“There is nothing to think, nothing to clutter my mind with. I do not yearn to think either. I leave my thoughts on my bed and come out a free soul.”

Another theme was that of the sense of safety that was experienced while listening to some environments and the sense of danger or threat induced by others. The sense of safety was associated with the natural environment:

“I feel a sense of control – I can move close to the birds or the waves and interact with it if I want to and only if I want to. Nothing in this environment is going to move in a way that may threaten my safety. I don’t have to be on my guard the whole time.”

“This makes me feel safe, I am not enclosed or locked up and I can control my actions and walk away if anything threatening occurs; there is an escape route. Also I feel safe because there are lots of bird calls. I guess this means that there is nothing overly threatening in my environment currently – if there was the birds would fly away or sound some bird alarm. They sound pretty contented and going about their lives so there must not be much to fear around.”

In contrast, the sound environment that was dominated by human pedestrians produced mixed responses with respect to people’s feeling of safety. Sometimes safety was enhanced:

“I feel almost slightly calm and almost like I’m waiting or walking at a leisurely pace. Nothing in this environment is threatening to me at all… Also if something bad occurs or something threatening occurs, I think my chances that people will come to my physical aid is high. I hear voices of both men and women of all ages – someone will be able to help me. Knowing that help is readily available also gives me a sense of peace.”

Or even a sense of it being so safe that it is dull:

“Generally this soundscape seems mundane and everyday. Sounds I am familiar with and not threatened by. Not quite peaceful, but certainly not annoying.”

Whereas sometimes there could be a mixed view of threats and excitement:

“On one hand, I feel anxious, I have associated crowds to danger and theft. I try my best to avoid them and the busyness of town while on the other hand, the busyness can become very exciting and positive, giving me a sense of adventure and disarray, a break from the boring routines I’ve inadvertently put in place in my life.”

And the same environment may be perceived as a threat:

“I find this environment quite loud and feel a bit nervous as to what is happening. The voices do not appear friendly and I feel unsafe. The motorbikes especially evoke a sense of fear and I don’t like them.”

On the other hand, the sound environment that was dominated by traffic was perceived only as threatening:

“I don’t feel safe at all – in fact I feel rather threatened. Passing traffic sounds are really close I’d much rather be a bit further away from them… the environment seems threatening and dangerous.”

“I find this environment quite loud and feel a bit nervous as to what is happening. The voices do not appear friendly and I feel unsafe. The motorbikes especially evoke a sense of fear and I don’t like them. I think there are lulls in the traffic which relax me, although at times I feel an accident could be imminent.”

Responses suggested that contemplation of sound environments could awaken spiritual feelings in people. The natural sounds were associated with spiritual uplifting:

“I want to find discover new things, and learn about the answers of life. I feel like I have so many questions and very little in the way of answers so far. Some questions I cannot even express, but I have a feeling of curiosity and hope that that feeling will take me somewhere should I act upon it.”

“So carefree, so full of spirit the chirping reverberates through the surrounding and penetrates the darkness. So happy, so light-hearted, bringing a sense of purity and innocence to this place.”

While the other soundscapes could be spiritually deadening:

“The whole drab affair is soul crushing when exposed to it for so long – need a change.”

“Why is there such a profound hate, hate I did not know I possessed. But yet it is there, it etches deep into me, scraping at my heart and pulling out ghosts which have been safely buried away.”

The sense of physical wellbeing was enhanced by some sound environments:

“I feel healthy – I’m awake early enough to hear the birds. […] I’m breathing fresh, unpolluted, virgin air.”

Whereas a sense of affliction was caused by others:

“I cannot get through, for there are too many people. The wait is giving me lines, a tight forehead, and I feel tired, very tired and a little short of breath.”

A sense of comfort and contentedness was associated with some soundscapes:

“Overall it’s pretty warm and cozy… ”

“I feel relaxed and a lot less on edge. The waves almost seem to lull me to sleep and the bird sounds are comforting.”

Whereas the traffic-dominated environment produced discomfort and the desire to escape:

“The air is dirty and I’m not comfortable. I feel like I’m heading for another long, restless and monotonous day in the office.”

“I can feel myself trying to leave my own body. Withdrawing just to escape the screams and roars.”

“I look to escape. I do not want to be here. I want us to be away from the city. Why did we create this? Is this necessary? Can’t it go back to the way it was before?”

In summary, the qualitative analysis generated eight themes related to the quality of the sound environment and nine themes related to the deeper internal response to it (Table 1). These themes, and the polar terms used to capture our understanding of each theme in semantic differential scales were used in the questionnaire. However, this process was not straightforward because (as pointed out earlier) the distinction between the perception of a sound and the emotions it evokes are not straightforward. To attempt to address this, and in order to provide a sense of the meaning intended for each scale, sets of semantic markers were used as seemed best subjectively. This meant that we used terms which were not necessarily in simple opposition to each other, and which potentially described different aspects of a theme. For example, the theme related to ‘Space’ was labeled ‘spacious/liberating/vast’ at one end of the scale, and ‘congested/claustrophobic/enclosed’ at the other. The meanings of the terms used, and their overlap captured as best we were able our understanding of the meaning behind the themes identified.

**Table 1:** Themes and terms used on each pole of the scale in the questionnaire.

Theme	Lower pole	Upper pole
**Qualities of the sound**		
Level	Very soft	Very loud
Pace	Leisurely	Fast
Clarity	Clear/distinct	Unclear/blurred/disorderly
Complexity	Simple sounds	Complex sounds
Space	Spacious/liberating/vast	Congested/claustrophobic/enclosed
Tone	Harmonious/melodious	Discordant/harsh
Stability	Dynamic/changing/up-and-down	Monotonous/in the same manner/flat
Pattern	Rhythmic/predictable	Irregular/random
**Feelings and emotions**		
Stimulation	Soothing/hypnotic	Arousing
Connection	A sense of belonging	A sense of alienation
Stress	Relaxation/tranquility/peace	Stress/anxiety annoyance/anger
Familiarity	Familiar/usual	Novel/unusual
Cognitive Load	Refreshed/rejuvenated	Distracted/mentally overloaded
Safety	Safe/a sense of control	Threatened/fearful
Spirit	Uplifted/meditative/transcendent	Oppressed/depressed
Wellbeing	Healthy/wholesome	Affliction/infirmity
Comfort	Contented/comfortable	Desire to escape/uncomfortable


## Phase 2: Quantitative Study

### Materials and Methods

The themes identified in Phase 1 were adapted to items in a questionnaire (Table 1 and Supplementary Material). There were 17 different themes identified by the qualitative analysis. Of these, two (Stimulation and Familiarity) appeared to have a multidimensional structure (see Qualitative Results), and therefore two more items were introduced to the questionnaire to allow this to be captured, but these were little-used by participants and were dropped from analyses and not presented in the quantitative part of the Results. The semantic-differential items were introduced by a short passage of text reading: “*Please listen to the sounds around you and rate the sound environment and your response(s) toward it by circling a number (1–6) on the following scales. If the scale is irrelevant to you, tick the ‘not applicable’ box beside it.”*

We did not attempt to limit or differentiate the descriptive (denotative) and affective (connotative) items.

Typically, a seven-point bipolar rating scale has been used in semantic differential scale research. This allows for a range of responses while also allowing a respondent to adopt the midpoint of a scale as a ‘null’ option. It is important to include a null option where a particular soundscape may simply not cause a particular response in a person, but the null option is also a danger in that it allows a respondent to opt out of making a decision about the sound environment or their response to it and thus reduces the value of data collected. Recognizing the competing issues, we used a six-point rating scale with the null point presented as a separate tick box labeled ‘N/A’ for each scale. We believed that this would tend to preserve the usefulness of data while still allowing the null option in a position that required respondents to make a definite choice to select it.

Scales were generated with as much information for respondents as possible. Each scale had a heading that reflected a theme about the soundscape identified in the writing during the first phase, and the poles of each scale were anchored with at least one term which we agreed would capture that extreme of the scale in question. If possible, multiple terms were used on the principle that if the meaning of each term has variability in the mind of a respondent, the areas of meaning which overlapped between terms would specify the concept we were asking about more precisely (Table 1).

As the aim was to create a questionnaire that could be distributed and completed by members of the public, focus was placed on reducing the number of items where possible. Preliminary versions of the questionnaire were trialed among a small group of people to assess issues like clarity, readability and ambiguity. An iterative process allowed for refinement of the questionnaire.

Adults were stopped in the street and asked if they would be willing to answer the questionnaire. Of these, 228 agreed and their data are presented here. This was done in four different locations within the central city: a park (*N* = 12), a quiet shopping street with mixed pedestrian and light vehicular use (*N* = 50), a busy main street with a mixture of pedestrian and vehicular traffic (*N* = 48), and a street heavily used by buses and other vehicles with fewer pedestrians (*N* = 59). The sites were selected because we were interested to test whether the questionnaire would provide different responses in quite subtly varying sound environments (i.e., the different types of street), and in a qualitatively different environment (the park).

In preparation for the analysis (SPSS v25), a check and reorganization of the variables was carried out. Questionnaire items were presented in random order to encourage respondents to read each question and provide thoughtful answers. To align the direction of responses with the label used for each theme, reverse coding was performed prior to analysis. This was for the items: Clarity, Space, Tone, Pattern, Connection, Familiarity, Safety, Spirit, Well-being, and Comfort. Follow-up items: ‘Type of Arousal’ which sought to operationalize the difference between feeling aroused in the sense of being excited and aroused in the sense of being overwhelmed; and ‘Feeling about familiar sounds’ which tried to operationalize the difference between comfortably familiar sounds and boring sounds were dropped from this analysis because they were conditional on prior items and this made interpretation difficult, as well as resulting in many missing data points.

A preliminary principle components analysis was conducted.

Principle axis factoring with obliminal rotation was used to assess the remaining 19 soundscape items. Obliminal rotation was preferred because it allows factors to be non-orthogonal (i.e., correlated), and there is no *a priori* reason to assume that soundscape factors would be orthogonal. Using the standard approach (i.e., the Kaiser criterion) of selecting factors with eigenvalues > 1, five factors were generated.

Internal reliability was assessed using Cronbach’s alpha. Since factor scores are based on all the items in the dataset, these values were generated based only on the raw scores for items with loadings >0.3 on each factor.

Mean factor scores for each of the five factors were compared between those responding in the park and in the different types of street using ANOVA. Distributions of scores within each area were checked for violations of normality and homoscedasticity assumptions and found to be acceptable. *Post hoc* least-significant difference tests were conducted: no attempt was made to control for ‘Type-1’ errors on the basis that the study was essentially exploratory, and therefore such errors would be less important than Type-2 errors which are more prevalent when using controlled testing.

### Results

**FIGURE 1:**
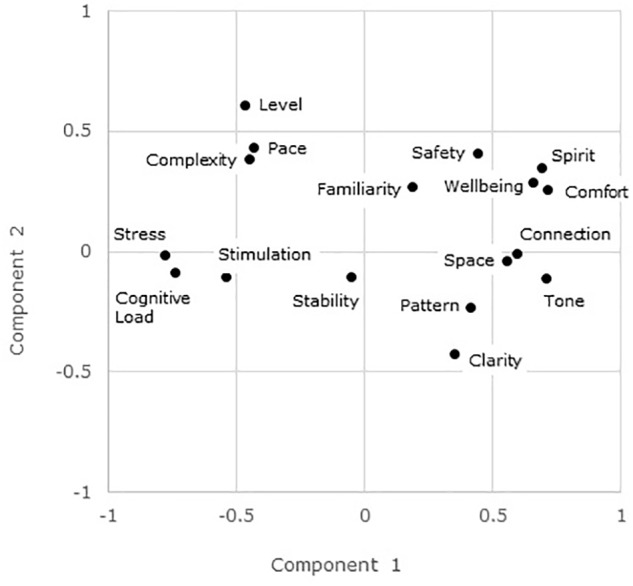
Loadings of the 17 items on each of the first two principle components.

The principle components analysis showed that there were five principle components with eigenvalues greater than one. The loadings of the 17 items on the first two components are plotted in Figure 1.

**FIGURE 2:**
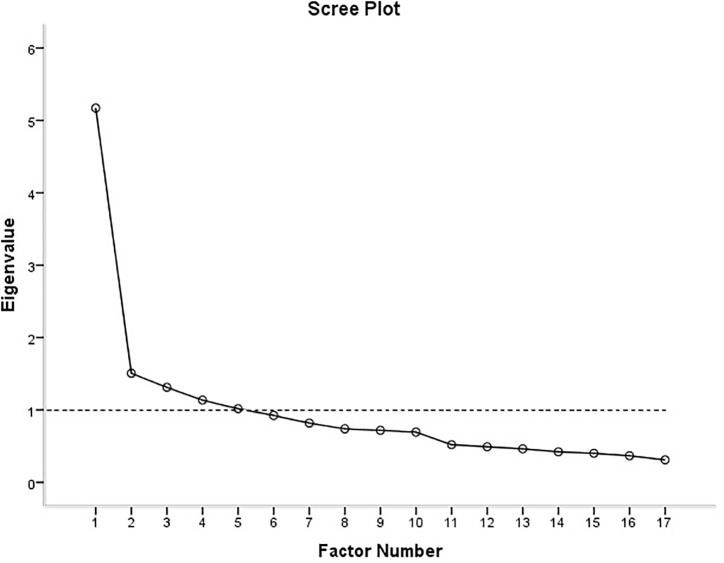
Scree plot showing eigenvalues obtained for factors obtained.

The sample was found to be suitable for factor analysis on the basis of a Kaiser–Meyer–Olkin score of 0.85 and a significant Bartlett’s Score. Five factors had eigenvalues greater than one (Figure 2). These explained 62% of the variance: Factor 1 explained 37%, and Factors 2–5 explained 9–6%, respectively.

The rotated solution with five factors explained 47% of the variance based on the extraction sums-of-squares loadings. Absolute factor loadings of greater than 0.3 were used to characterize each factor (Table 2).

**Table 2:** Factor loadings for each of the 17 soundscape items.

	Factor names				
	Calming	Protecting	Hectic	Belonging	Stability
Level			0.777		
Pace			0.528		
Clarity			–0.439		
Complexity					
Stimulation	–0.553				
Space	0.451				
Tone	0.715				
Stability					0.706
Pattern	0.324				
Connection				0.638	
Stress	–0.616				
Familiarity				0.489	
Cognitive Load	–0.697				
Safety		0.728			
Spirit	0.485	0.516			
Wellbeing		0.642			
Comfort		0.566			


*Only loadings of greater than 0.3 are shown to facilitate the visualization of the factors.*

Stimulation, Stress, and Cognitive Load loaded negatively and Space, Tone, Pattern, and Spirit loaded positively on a factor that was labeled ‘Calming.’ A person who found a soundscape ‘Calming’ would tend toward the descriptors: ‘soothing/hypnotic, spacious/liberating/vast, harmonious/melodious, rhythmic/predictable, tranquility/peaceful, refreshed/rejuvenated, and uplifted/meditative/transcendent.’ Cronbach’s alpha score for this scale was 0.81.

Safety, Spirit, Wellbeing, and Comfort loaded positively on a factor labeled ‘Protecting.’ A person who felt a sense of being ‘Protected’ in the soundscape would tend to use the descriptors: ‘safe/a sense of control, uplifted/meditative/transcendent, healthy/wholesome, and contented/comfortable.’ Cronbach’s alpha score for this scale was 0.78.

Level and Pace loaded positively and Clarity negatively on a factor that was labeled ‘Hectic,’ capturing as it did, loud, quickly changing, and unclear sounds. A person who found a soundscape ‘Hectic’ would tend toward the descriptors: ‘very loud, fast, and unclear/blurred/disorderly.’ Cronbach’s alpha score for this scale was 0.60.

Connection and Familiarity loaded together and positively on a factor labeled ‘Belonging.’ A person who felt a sense of belonging to the soundscape would tend to use the descriptors: ‘a sense of belonging, and familiar/usual.’ Cronbach’s alpha score for this scale was 0.39.

Stability alone had a high loading on the fifth factor, and thus this factor was labeled ‘Stability.’ A person who scored high on this scale would have used the descriptors ‘monotonous/in the same manner/flat’ to describe the soundscape.

Complexity did not produce sufficiently large loadings on any of the factors to be considered in the naming of factors, suggesting that its loading was distributed rather evenly across the factors. Considering cross-loading, only Spirit loaded > 0.3 on more than one factor: it was represented in both the Calming and Protecting factors.

Oblique factor analysis allows factors to correlate. In most cases, correlations were small (<0.3), however, Calming and Protected correlated moderately (*r* = 0.47), as did Calming and Hectic (*r* = -0.43). Given the nature of these factors, and the observation that Spirit loaded highly on both Calming and Protecting, the correlations and their directions are not unexpected.

**FIGURE 3:**
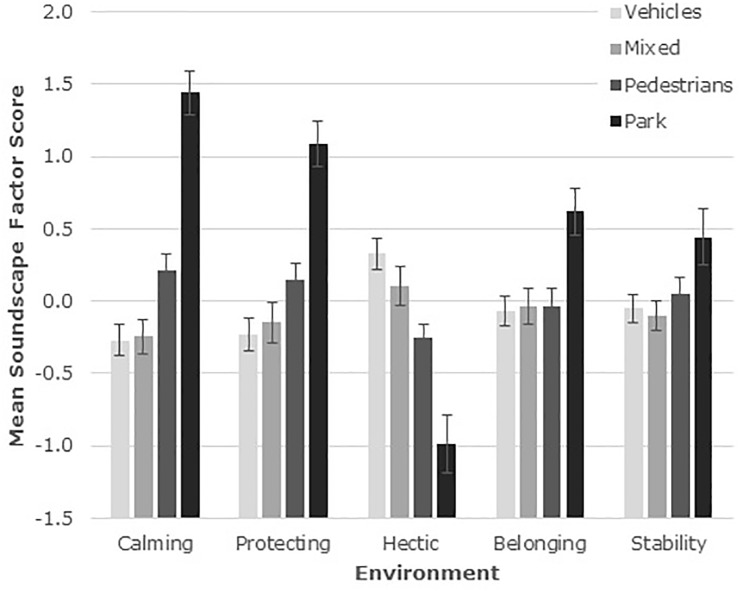
Mean Soundscape Factor Scores for people responding in the four city environments. Scores on each of the five factors are represented separately. Error bars represent one standard error of the mean.

Factor Scores were generated and the scores were compared between those who completed the questionnaire on city streets and those who completed it in a park (Figure 3). Negative scores indicate that, on average, people experienced the opposite of the factor name, and positive or negative scores further from zero reflect the degree that each factor was experienced.

Analyses were conducted to compare the responses across the four environments (Figure 3). This used General Linear Modeling with the four types of area (street dominated by vehicles, mixed, street dominated by pedestrians, and park) as the independent variable and the five Factors as dependent variables. The Calming [*F*(3,168) = 17.25, *p* < 0.001], Protecting [*F*(3,165) = 9.09, *p* < 0.001], and Hectic [*F*(3,168) = 11.14, *p* < 0.001] factors were clearly significantly different between areas. Belonging [*F*(3,168) = 2.56, *p* = 0.056] and Stability [*F*(3,168) = 1.86, *p* = 0.14] differed more marginally. The direction of effects was consistent: Calming, Protecting, Belonging, and Stability were higher, and Hectic was lower for the park than for the street environments. *Post hoc* testing (Least Significant Differences) showed that all the soundscape factors differed between the park environment and at least some of the street environments. Calming was highest in the park and was also higher in the pedestrian-dominated street than in either the mixed or vehicle street types. Protecting was higher in the park than all the street types and was also higher in the pedestrian-dominated than the vehicle-dominated street. Hectic was lower in the park than any of the streets and was also lower in the pedestrian-dominated street than in either the mixed or vehicle-dominated street. Belonging was higher in the park than any of the streets, which did not differ between each other. Stability was higher in the park than in any of the streets apart from the pedestrian, and no other differences were observed (Figure 3).

## Discussion

We exposed people who self-identified as expressive writers to different sound environments and asked them to write about their reactions. The writings were then subjected to a qualitative, thematic analysis and the themes which emerged were used as the basis for a seventeen-item questionnaire. This was administered to passers-by in Auckland City. The items generated by the thematic analysis could be rendered down to five factors which underlay the responses made by people to them: Calming, Protecting, Hectic, Belonging, and Stability.

The themes extracted from the expressive writing part of the research (i.e., Phase 1) were broadly consistent with the concepts used in other similar studies. For example, the set of items used for urban soundscapes by Kang and Zhang (2010) and since used in other studies included equivalent concepts to the themes of level, pace, complexity, tone, stress, and wellbeing. On the other hand, our writers came up with other themes that did not appear in that set (Table 1). Comparison with other, much larger sets of terms (Axelsson et al., 2010) shows similar parallels and discrepancies. The process of seeking to capture the elements of the soundscape is not straightforward, and the use of different approaches for this crucial first step is important. Overall, after rotation, our five factors explained 47% of the variance in the data we collected. This is similar to the 53% reported in the other study that used a similar approach of combining descriptive and affective items to describe urban spaces (Kang and Zhang, 2010).

Our questionnaire was based on the themes identified in the qualitative writing phase of the research. It was useable by the general public and showed patterns in the results consistent with previous research suggesting that parks would differ from urban streets in being calmer, more protecting and less hectic (Carrus et al., 2017). Our questionnaire was not, however, very useful for discriminating between soundscapes associated with city streets that had differing degrees of heavy vehicular traffic use. Saying this, differences were observed in some factors between a pedestrian-dominated street environment (with light vehicular use) and streets that had either mixed or predominately vehicular usage. The pedestrian-dominated environment was more Calming and less Hectic than the others. The capacity of the questionnaire to differentiate between these environments, provides some support for the validity of the measure. Though it is not possible to validate psychometric scales like these absolutely, the capacity to detect statistical differences associated with qualitatively different environments supports the idea that the scales had validity.

We assessed the internal reliability of the scales, and these were generally at acceptable levels (approximately 0.8) for scales with several items: scales with few items will tend to show lower measures of internal reliability, so lower values in these do not imply poor reliability. Use of the scales in similar urban environments (i.e., the different types of street) showed only slightly different mean scores, which implies that there is some reliability in responses given the similar environments. It would also be interesting to test reliability over time in the same location, however, this would be difficult given that factors influencing the soundscape might change, so careful characterization of the environments would be important to allow any variation to be clearly indicated as either due to changing environment or to unreliability of the measure.

The generalizability of the results is questionable because our sample was quite small, and depended on voluntary involvement of passers-by in public streets rather than carefully conducted random sampling. Saying this, we were not seeking to provide a definitive set of data about the soundscape in Auckland City. Rather, we were seeking to test whether people could and would respond to the questionnaire, and if so whether there was some meaningful structure to the way they responded. We believe there was and so are comfortable that the research supports the approach as a way of generating data.

Soundscape research such as ours seeks to quantify a subjective judgment. As researchers, we hope that this is possible because there is an element of consistency in the sound environments, and since people are all from the same species, it would be likely that there would be commonality in the factors that drive us to experience different feelings (Andringa and Lanser, 2013). The lack of consistency in subjective judgments depends on many factors. We propose that there are multiple loosely coupled systems in operation to explain individual responses to questions about the soundscape:

(1) The set of concepts and emotions that each person associates with each lexical item will vary according to their experiences and idiolect: a word will not necessarily capture the same meaning for everyone. Furthermore, for a person to express an emotion via a closed-set questionnaire such as we used in Phase 2 of this study, there must be some item present that would capture the meanings that person perceives; if there is not, then that internal state would go unreported.
(2) The emotional response to a given sound environment will differ depending on a person’s understanding of the situation, their previous experiences, personality, and current state of mind. Furthermore, the pallet of affective states that different people experience may vary according to their experiences and psychophysiological make-up.
(3) Definitions of the soundscape explicitly accept interactions with other sensory information. The sound environment is highly integrated with other information in our minds. While the field has acknowledged this, it still presents an unquantified element in the coupling between the sound environment and the soundscape.
(4) The emotional states that people experience in response to sounds may be hard to distinguish from their experience of the sound itself. The concepts of sound in the sense of physical pressure fluctuations and sound as a percept may be intertwined in the mind. In other words, people know how they feel and what the world is like, but they do not necessarily separate these two sets of concepts. An example of this is the Calmness factor, which combined denotative (Space, Tone) and connotative (Stimulation, Stress, Cognitive Load, Spirit) items. It would be convenient if these were separate in the minds of people, but our results demonstrate that they are not cleanly separated. Other factors appeared to display organization along denotative and connotative lines: Protecting and Belonging appeared connotative, and Hectic and Stability were apparently more denotative in terms of the highly loaded items (Table 2). Even in these cases though, our qualitative analyses had already revealed that, for example, ‘pace,’ which was loaded into the Hectic factor, seems to reflect not only the denotative temporal quality of the sound environment, but also reflects a connotative adaptation of the internal state of listeners in response to this.

Together, these four issues combine to reduce a researcher’s capacity to gain a full understanding of a person’s perception of the sound environment. Accepting this requires us to put aside some of our tightly organized, analytical thinking at one level while maintaining it very carefully at another.

We identified five factors on the basis of eigenvalues. In factor analysis, there is no strict rule for deciding on the factor structure, and a structure with less factors is generally preferable on the basis that it can be imagined as a space (if there are three or less dimensions), or even drawn. Some soundscape studies have identified factor structures on the basis of eigenvalues and then dropped factors which the authors feel do not contribute much to the understanding of the data (e.g., Axelsson et al., 2010). This is a perfectly acceptable practice. We chose to preserve even the fifth factor (Stability) which had a strong loading from only one item. This might be regarded as improper on the basis that factor analysis is valuable because it reduces the number of dimensions below the original, and this is the basis for the Kaiser criterion that factors should have eigenvalues greater than one to be regarded as efficient. Nonetheless, we felt that it was justified. Firstly, the item ‘Stability’ did not load much on any other factor and it was identified as relevant in the writing. Secondly, in principle, the soundscape is still poorly understood so we felt that any contributing factor should not be neglected until the provision of evidence to the contrary. Thirdly, it must be remembered that in factor analysis the relative ‘strengths’ of the factors is somewhat arbitrary. The unfactorized data may be envisaged as an N-dimensional cloud, where N is the number of items in the questionnaire. Commonality in the alignment of underlying meanings of the items in the minds of respondents would tend to reduce the dimensionality of the cloud due to the tendency for correlations between responses to those items, and thus N can be reduced while losing only slight variations in the cloud’s dimensions. However, we do not know what the true dimensionality of the soundscape is and our choice of items is thus rather arbitrary. The finding that only one item substantially loaded on the Stability factor, and that the factor explained 6% of the variance in the items used does not tell us that it is unimportant. It only tells us that Stability does not relate much to the other items we have chosen.

Factor analysis therefore allows the grouping of items which are originally separate. It provides a simplification of data but the process of naming the factors also adds to the understanding of the underlying influences on the data. The factor we labeled ‘Calming’ implies that soundscapes that were harmonious, following a pattern, and providing a sense of spaciousness were associated with people feeling soothed and tranquil; and that this was rejuvenating and enabled spiritual transcendence. This picture is helpful in that it seems to follow from descriptive features to affective states and then goes beyond simple emotions into higher aspects of our being. The implication is that soundscapes can influence us very deeply, and this dimension is consistent with the dimension ‘Calmness’ (Cain et al., 2013).

Similarly, the factor we named ‘Protecting’ captured the idea that soundscapes in which people felt safe provided contentment and in such soundscapes people felt both physically wholesome and spiritually uplifted. Again, the depth of the concepts drawn from the writing produced items which allowed respondents to express the deep impact of feelings beyond simple emotions and provides a picture of the unfolding influences of higher-level cognition. Usefully, this level of responding was accessible from passers-by in the street who took only moments to reflect. The idea that the acoustic environment feeling protected is important for people, and theoretical work has been done in this direction (van den Bosch et al., 2016). The emergence of a factor that relates directly to this suggests that the approach used may provide a useful model for soundscape research into improving the soundscape via interventions.

We used the term ‘Hectic’ to label another factor because it captures the idea of loud and low fidelity environments causing people to feel hurried and pressured temporally. The relationship of time to soundscapes has been considered previously (e.g., Kang and Zhang, 2010). Time is a physical dimension within which we have no control. Nonetheless, as people, we feel that our relationship with time varies, speeding up and slowing down depending on the conditions and our state of mind. The soundscape appears to contribute to this, and the associations described by this factor provide some sense of how. More understanding of how and why the soundscape contributes to this would be important.

The other two factors we identified had high loadings from only two and one item, respectively. ‘Belonging’ combined the idea that a person could feel familiar with a soundscape and that this would be associated with a feeling of belonging to it. Interestingly, everyone surveyed must have been familiar with the soundscapes dominated by traffic and other pedestrians, but nonetheless the responses did not reflect this: rather the sense of both familiarity and belonging was greater in the park. Connection and Familiarity loaded together and positively on a factor labeled ‘Belonging’ and this may partly correspond to the factor labeled ‘Familiarity’ in previous research (Axelsson et al., 2010). We named the fifth factor ‘Stability’ and it was associated with a less varying soundscape, which people observed in the park more than in the vehicle-dominated streets. As we argued above, this factor might explain some important aspect of the soundscape for which we have no good theoretical understanding. Of course, it may relate to the concept of ‘Eventfulness’ (Axelsson et al., 2010) or ‘Vibrancy’ (Cain et al., 2013), which have also been identified as being important both from a theoretical perspective (Andringa and Lanser, 2013; van den Bosch et al., 2018). The structure of the factor analysis here may be driven partly by the lack of an interestingly eventful soundscape in the areas where we administered the questionnaire: streets and a park, none of which have much vibrancy or many events occurring. Future research using the questionnaire in areas with more interesting and relevant sounds might produce a different structure.

The research has other limitations. To us, the most significant caveat is that during the creative writing phase we used sound exposures in the absence of other (visual, olfactory, etc.) stimulation which may have influenced the experience of the soundscapes. We wanted the participants to focus on the sound so that their writing would capture those aspects of the soundscape for us to use in the development of the questionnaire. We thought that adding other sensory information alongside the sound, or asking participants to conduct the writing exercise in the real world would have provided distractions from the acoustic aspects of the environment to which we very much wanted them to attend. It is possible that the themes may have been broader had the writing been conducted in multisensory environments, but the positive aspect of running the study the way we did was that we found few references to non-auditory aspects of the virtual environments in the writing. Nonetheless, the seventeen themes identified in the qualitative phase of the research may have underestimated the potential themes in soundscapes, and thus is it worth considering whether more may be valuable.

The sound environments where we administered the questionnaires were reasonably similar, apart from the park, and it may be interesting to test the questionnaire in a more widely varying set of environments. Furthermore, the particular range of four environments we used might have introduced patterns to the data that could have led to the factor structure being different from what it would have been if we had included other environments. Future research in which we increase the number of environments may well alter the factor structure observed. Finally, we did not assess the extent to which the questionnaire would detect changes in an environment, and an interesting area for future research is to administer it in a longitudinal manner throughout a period of change in a sound environment such as a redevelopment of an area of the city.

Some excellent theory and research has moved us toward a unifying theory to explain the various findings from soundscape research. Good theory can help direct research and allow more specific hypotheses to be tested. We regard our present work as largely exploratory and aimed at stimulating ideas about possible directions for growth in existing theories. We have tried to demonstrate that there are possibly more complexities to the soundscape than are captured by our two-dimensional models and to remind researchers that the items used to generate factors are crucial, because they dictate the entire conceptual space which then provides the components of the theory. With a different theoretical stance, the descriptors used in questionnaires would change, and thus the apparent factors that emerge from them would be different. An example of another way of considering the interplay between our senses, cognition, and emotions is that of a valuation-based process wherein we evaluate environments based on a complex internal model that would weigh up the survival benefits of a given environment and take into account factors such as the opposing principles of competition and social support from other people (Mercado-Doménech et al., 2017). By thinking more about the ways that people feel due to their experience of a soundscape, why they would feel this, and crucially, how they describe those feelings, we may move toward a more complex model and assessment of the soundscape.

We did not include previously used scales alongside our one for comparison, and this would be interesting to do in a future study. Nonetheless, it is interesting and useful to speculate about the possible correspondences between factors identified in different studies. We have mentioned above that Calming and Belonging seem to correspond, at least in part, with factors identified in earlier work. The factor we labeled ‘Protecting’ might correspond to earlier-identified ‘Pleasantness,’ and ‘Hectic’ might correspond to ‘Eventfulness.’ Stability could perhaps represent the other pole of factors that have been labeled ‘Excitingness’ or ‘Vibrancy.’ Published research with semantic differential scales and in similar sound environments (outdoor, urban) to those we used produced a pattern of responses that was somewhat similar to ours (Kang and Zhang, 2010). The earlier research identified four factors: relaxation, communication, spatiality, and dynamics. Our factor label ‘Calming’ sounds similar to the earlier ‘Relaxation,’ though our version did not include large loadings from our items ‘comfort’ or ‘level’ which appear to correspond to the items ‘comfort-discomfort’ and ‘quiet-noisy’ in the earlier study, while the other items in the relaxation factor did not have equivalents in our questionnaire. This may imply that the similarity in the factor name is rather superficial. The second and third factors in the earlier study were labeled ‘Communication’ and ‘Spatiality,’ and neither the names nor the items that load on these factors appear to correspond to factors we observed. On the other hand, the earlier study’s fourth factor ‘Dynamics’ might possibly correspond to our ‘Hectic’ in that the scales ‘hard-soft’ and ‘fast-slow,’ which loaded highly on it might be similar to the experiences captured by our ‘level,’ ‘pace’ and ‘clarity’ items. It has been suggested previously that Kang and Zhang’s ‘calming’ and ‘dynamics’ factors may correspond to the commonly reported two-dimensional soundscape structure (Davies and Murphy, 2012). If so, then the possible similarities with our work may support the notion of these two dimensions.

We encourage caution with respect to the factor structure we have described; the research was done to develop and field-test a questionnaire, and in this respect we feel it was successful. However, our data were captured in a limited set of sound environments, and this would limit the scope of the factors that we could possibly identify, while potentially introducing spurious correlations that might have led to apparent factors that would not be present in more representative datasets. As more research is conducted and theory generated, greater understanding of the range of aspects of the soundscape will emerge.

## Conclusion

We believe that the approach of bringing creativity to the initial set of soundscape-related items was useful. It provided both similarities and differences with previous research, and the questionnaire was workable in principle, with measureable differences in soundscapes between different urban sound environments. It allowed ordinary people stopped in the street to provide complex and deep responses about the impact of the soundscape on themselves, and it might be useful for those such as acousticians, architects, and planners trying to influence soundscapes. From a scientific perspective, some of the aspects of soundscapes that are suggested by the research may open up interesting directions for more research and the development of theory. We hope to develop the techniques used here further, and to test the questionnaire in more differing environments.

## Author Contributions

DW wrote the first draft of the paper, contributed to the design of the research and the qualitative analyses, was responsible for the quantitative data analyses, and contributed to the interpretation of the data. DS and KD contributed to the writing of the paper and the interpretations of the data. MT contributed to the writing of the paper and the interpretations of the data, collected part of the data, and contributed to the design of the research and the qualitative analyses. GC contributed to the writing of the paper and the interpretations of the data and collected most of the quantitative data.

## Conflict of Interest Statement

The authors declare that the research was conducted in the absence of any commercial or financial relationships that could be construed as a potential conflict of interest.

## Supplementary Material

Table S1 Soundscape questionnaire.
