# Supplementary material for: Use of Creative Writing to Develop a Semantic Differential Tool for Assessing Soundscapes
Source: Front Psychol. 2019 Feb 5;9:2698. doi: 10.3389/fpsyg.2018.02698 (PMC6370738; doi:10.3389/fpsyg.2018.02698)
Supplement: Table S1 — Soundscape questionnaire. [file Table_1.docx]

**Soundscape Questionnaire**

*Please listen to the sounds around you and rate the sound environment and your response(s) towards it by circling a number (1-6) on the following scales.*  *If the scale is irrelevant to you, tick the ‘not applicable’ box beside it.*

| **Soundscape feature/**  **your response** |  | |  |  |  |  |  |  |  | **Not applicable (tick)** |
| --- | --- | --- | --- | --- | --- | --- | --- | --- | --- | --- |
|  | **Very soft** | |  |  |  |  |  |  | **Very loud** |  |
| *Overall level* |  | | **1** | **2** | **3** | **4** | **5** | **6** |  |  |
|  |  | |  |  |  |  |  |  |  |  |
|  | **Leisurely** | |  |  |  |  |  |  | **Fast** |  |
| *Pace* |  | | **1** | **2** | **3** | **4** | **5** | **6** |  |  |
|  |  | |  |  |  |  |  |  |  |  |
|  | **Clear/**  **distinct** | |  |  |  |  |  |  | **Unclear/ blurred/ disorderly** |  |
| *Clarity* |  | | **1** | **2** | **3** | **4** | **5** | **6** |  |  |
|  |  | |  |  |  |  |  |  |  |  |
|  | **Simple**  **sounds** | |  |  |  |  |  |  | **Complex**  **sounds** |  |
| *Complexity* |  | | **1** | **2** | **3** | **4** | **5** | **6** |  |  |
|  |  | |  |  |  |  |  |  |  |  |
|  | **Soothing/**  **hypnotic** | |  |  |  |  |  |  | **Arousing** |  |
| *Stimulation* |  | | **1** | **2** | **3** | **4** | **5** | **6** |  |  |
|  |  | |  |  |  |  |  |  |  |  |
|  | **Spacious/**  **liberating/**  **vast** | |  |  |  |  |  |  | **Congested/ claustrophobic/ enclosed** |  |
| *Space* |  | | **1** | **2** | **3** | **4** | **5** | **6** |  |  |
|  |  | |  |  |  |  |  |  |  |  |
|  | **Harmonious/ melodious** | |  |  |  |  |  |  | **Discordant/ harsh** |  |
| *Tone* |  | | **1** | **2** | **3** | **4** | **5** | **6** |  |  |
|  |  | |  |  |  |  |  |  |  |  |
|  | **Dynamic/ changing/ up-and-down** | |  |  |  |  |  |  | **Monotonous/ in the same manner/ flat** |  |
| *Stability* |  | | **1** | **2** | **3** | **4** | **5** | **6** |  |  |
|  |  |  | |  |  |  |  |  |  |  |
|  | **Rhythmic/ predictable** |  | |  |  |  |  |  | **Irregular/ random** |  |
| *Pattern* |  | **1** | | **2** | **3** | **4** | **5** | **6** |  |  |
|  |  |  | |  |  |  |  |  |  |  |
|  | **A sense of belonging** |  | |  |  |  |  |  | **A sense of alienation** |  |
| *Your connection to the soundscape* |  | **1** | | **2** | **3** | **4** | **5** | **6** |  |  |
|  |  |  | |  |  |  |  |  |  |  |
|  | **Relaxation/**  **tranquillity/ peace** |  | |  |  |  |  |  | **Stress/ anxiety annoyance/ anger** |  |
| *Relaxation/ stress* |  | **1** | | **2** | **3** | **4** | **5** | **6** |  |  |
|  |  |  | |  |  |  |  |  |  |  |

|  | **Familiar/**  **usual** |  |  |  |  |  |  | **Novel/ unusual** |  |
| --- | --- | --- | --- | --- | --- | --- | --- | --- | --- |
| *Familiarity* |  | **1** | **2** | **3** | **4** | **5** | **6** |  |  |
|  |  |  |  |  |  |  |  |  |  |
|  | **Refreshed/ rejuvenated** |  |  |  |  |  |  | **Distracted/ mentally overloaded** |  |
| *Cognitive load* |  | **1** | **2** | **3** | **4** | **5** | **6** |  |  |
|  |  |  |  |  |  |  |  |  |  |
|  | **Safe/ a sense of control** |  |  |  |  |  |  | **Threatened/ fearful** |  |
| *Your safety* |  | **1** | **2** | **3** | **4** | **5** | **6** |  |  |
|  |  |  |  |  |  |  |  |  |  |
|  | **Uplifted/ meditative/ transcendent** |  |  |  |  |  |  | **Oppressed/ depressed** |  |
| *Spirit* |  | **1** | **2** | **3** | **4** | **5** | **6** |  |  |
|  |  |  |  |  |  |  |  |  |  |
|  | **Healthy/ wholesome** |  |  |  |  |  |  | **Affliction/ infirmity** |  |
| *Your well-being* |  | **1** | **2** | **3** | **4** | **5** | **6** |  |  |
|  |  |  |  |  |  |  |  |  |  |
|  | **Contented/ comfortable** |  |  |  |  |  |  | **Desire to escape/ uncomfortable** |  |
| *Comfort* |  | **1** | **2** | **3** | **4** | **5** | **6** |  |  |
